# Supplementary figures and images for: TaRTLEt: Transcriptionally-active Riboswitch Tracer Leveraging Edge deTection
Source: PeerJ. 2025 May 26;13:e19418. doi: 10.7717/peerj.19418 (PMC12121620; doi:10.7717/peerj.19418)

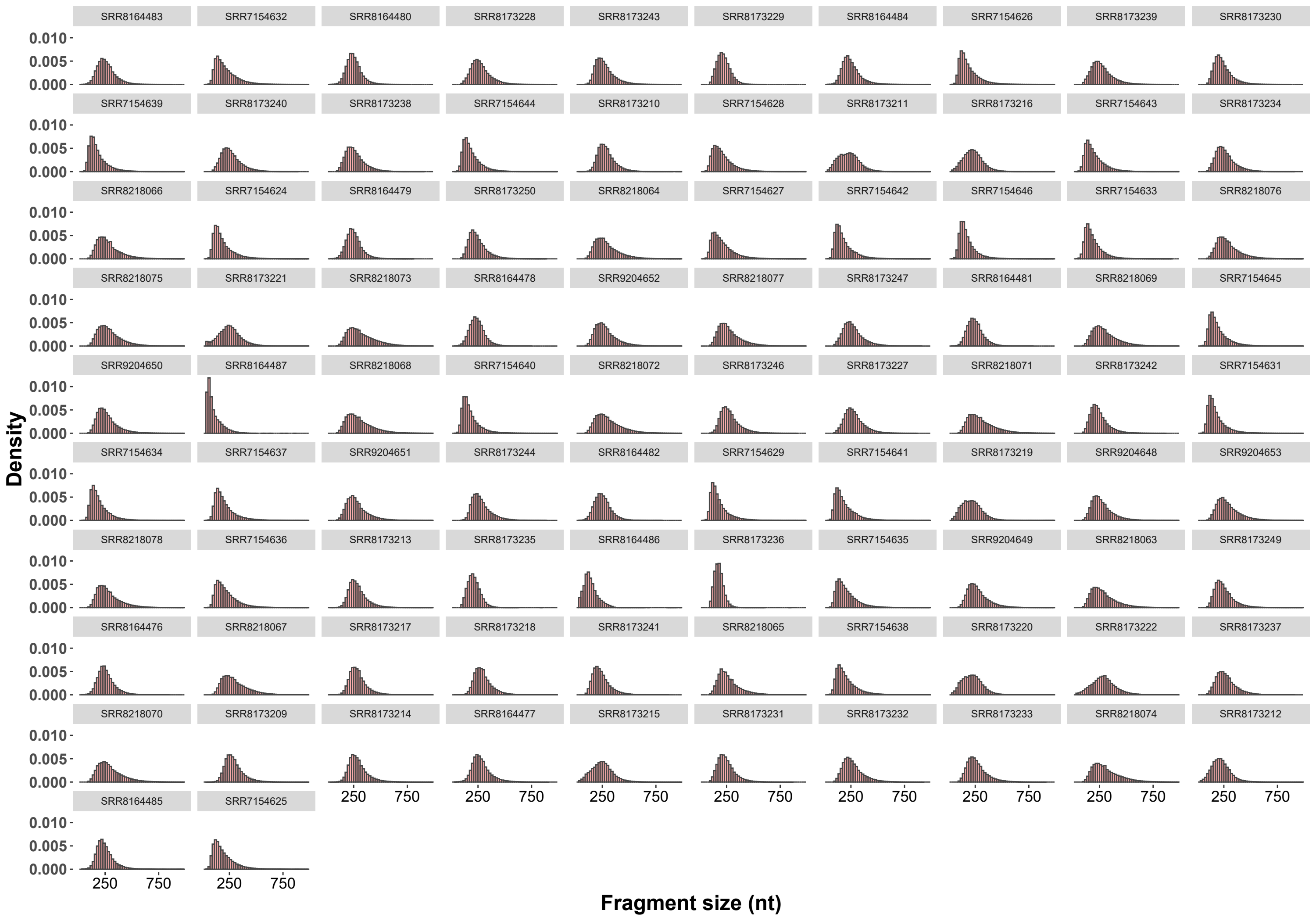

Supplement: Supplemental Information 1 [file peerj-13-19418-s001.pdf]

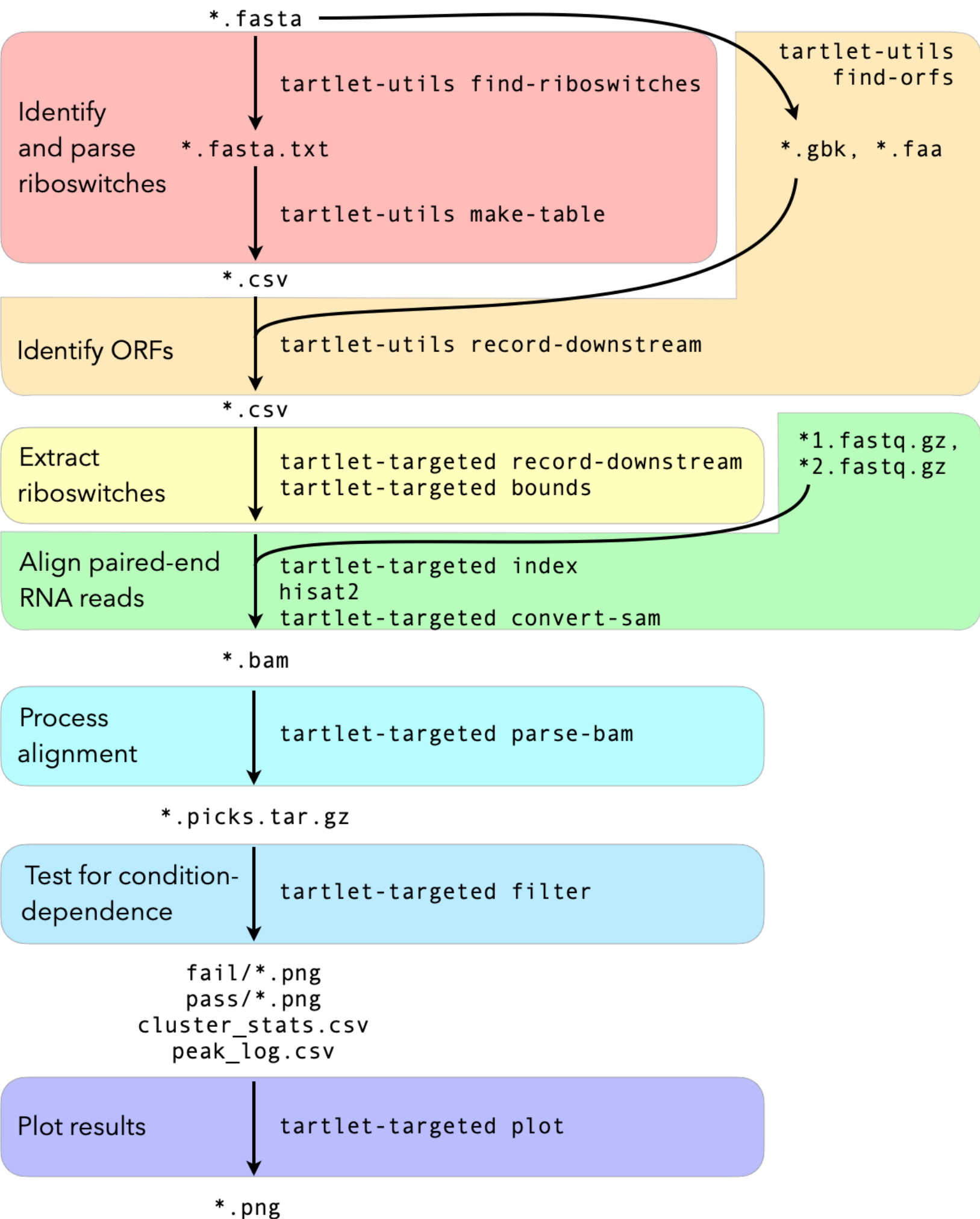

Supplement: Supplemental Information 2 — The TaRTLEt workflow requires (meta)genomic reference sequences (*.fasta) and (meta)transcriptomic paired-end RNA reads (*1.fastq.gz, *2.fastq.gz). Scripts used and output files produced are shown for each stage of processing. [file peerj-13-19418-s002.pdf]

## B. subtilis riboswitches across all available conditions

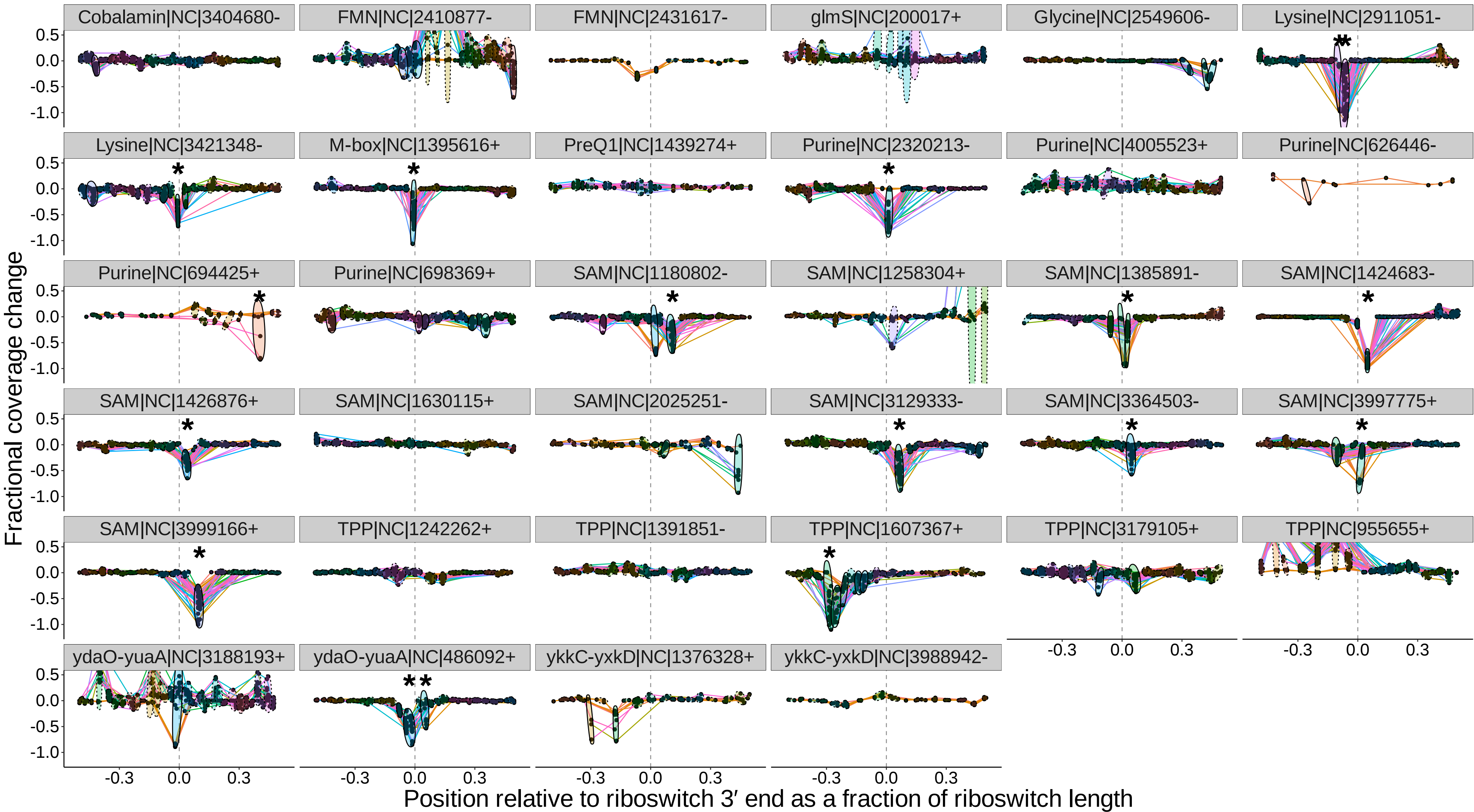

Supplement: Supplemental Information 4 — Peak clusters are grouped by colored ellipses; clusters with at least one significant transcription-termination peak are bounded by solid ellipses, and those with no significant peaks are bounded by dashed ellipses. Asterisks mark peak clusters that (i) contain at least one significant transcription-termination peak, (ii) have significantly lower mean (p < 0.05, Mann-Whitney one-tailed U-test) than the set of all other peak clusters, and (iii) have significantly higher variance (p < 0.05, Levene’s test) when compared to randomly sampled sets of the same number of peaks. [file peerj-13-19418-s004.pdf]

# B. anthracis riboswitches across all available conditions

Fractional coverage change

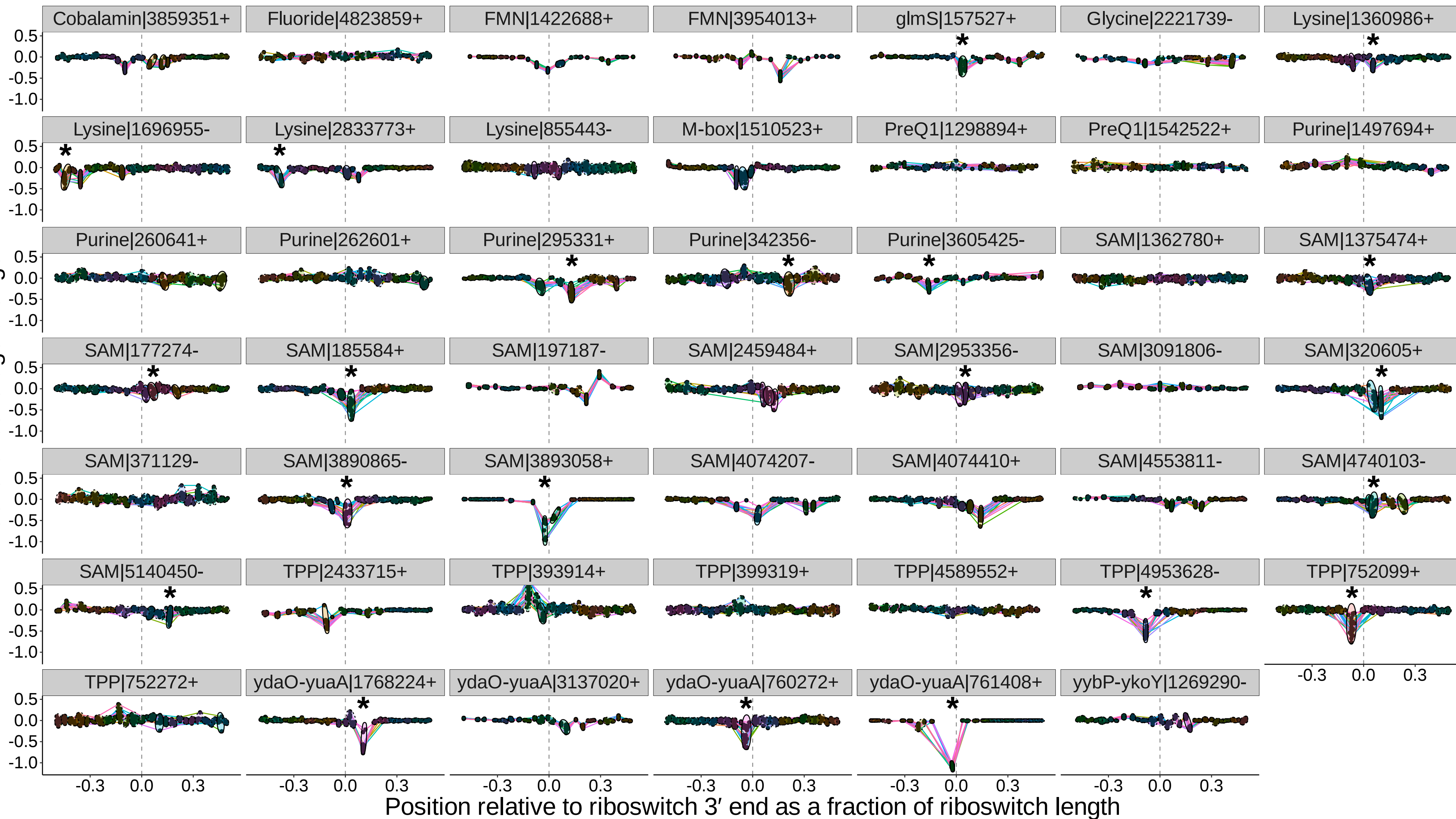

Supplement: Supplemental Information 5 — Peak clusters are grouped by colored ellipses; clusters with at least one significant transcription-termination peak are bounded by solid ellipses, and those with no significant peaks are bounded by dashed ellipses. Asterisks mark peak clusters that (i) contain at least one significant transcription-termination peak, (ii) have significantly lower mean (p < 0.05, Mann-Whitney one-tailed U-test) than the set of all other peak clusters, and (iii) have significantly higher variance (p < 0.05, Levene’s test) when compared to randomly sampled sets of the same number of peaks. [file peerj-13-19418-s005.pdf]

# E. faecalis riboswitches across all available conditions

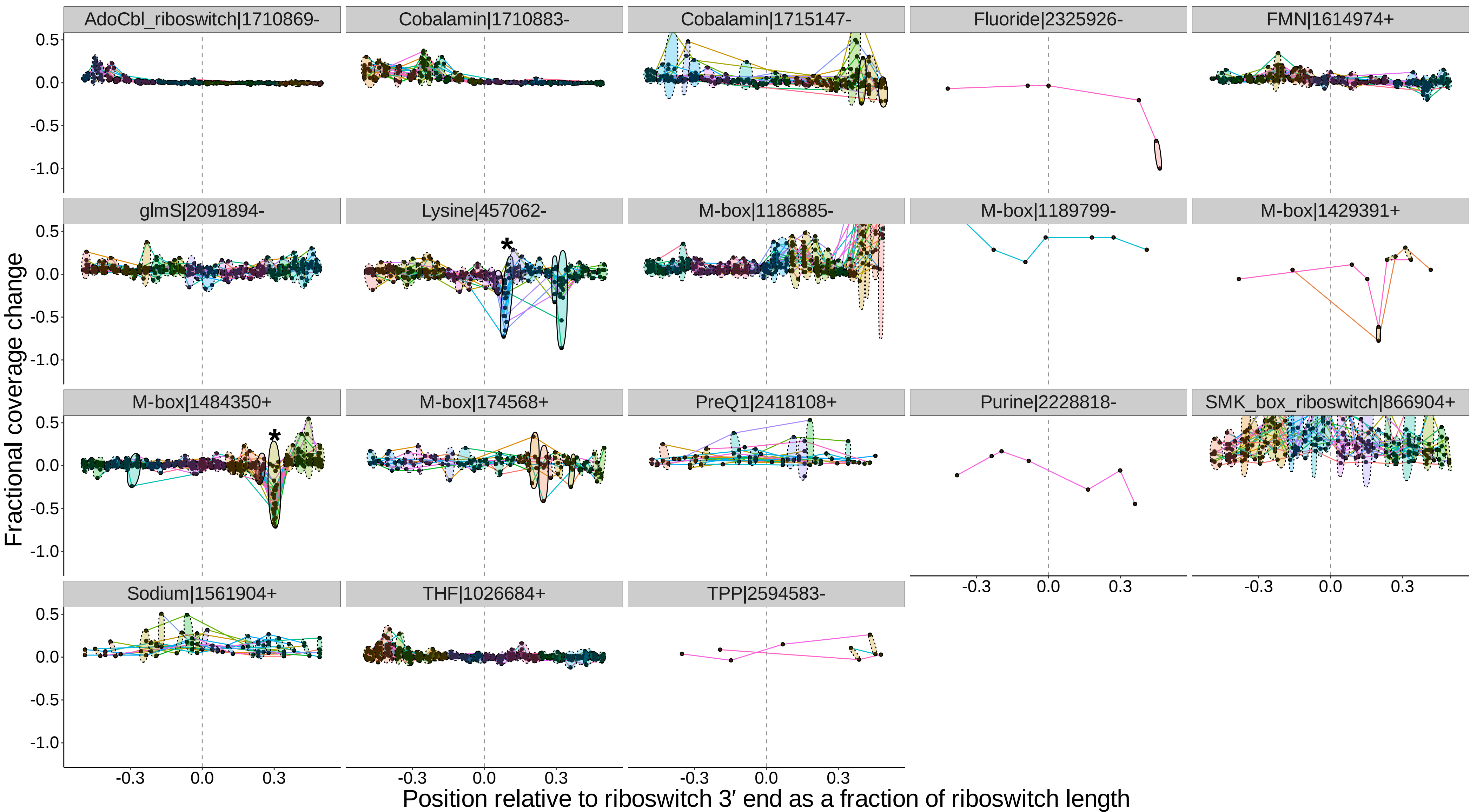

Supplement: Supplemental Information 6 — Peak clusters are grouped by colored ellipses; clusters with at least one significant transcription-termination peak are bounded by solid ellipses, and those with no significant peaks are bounded by dashed ellipses. Asterisks mark peak clusters that (i) contain at least one significant transcription-termination peak, (ii) have significantly lower mean (p < 0.05, Mann-Whitney one-tailed U-test) than the set of all other peak clusters, and (iii) have significantly higher variance (p < 0.05, Levene’s test) when compared to randomly sampled sets of the same number of peaks. [file peerj-13-19418-s006.pdf]

*S. enterica* riboswitches across all available conditions

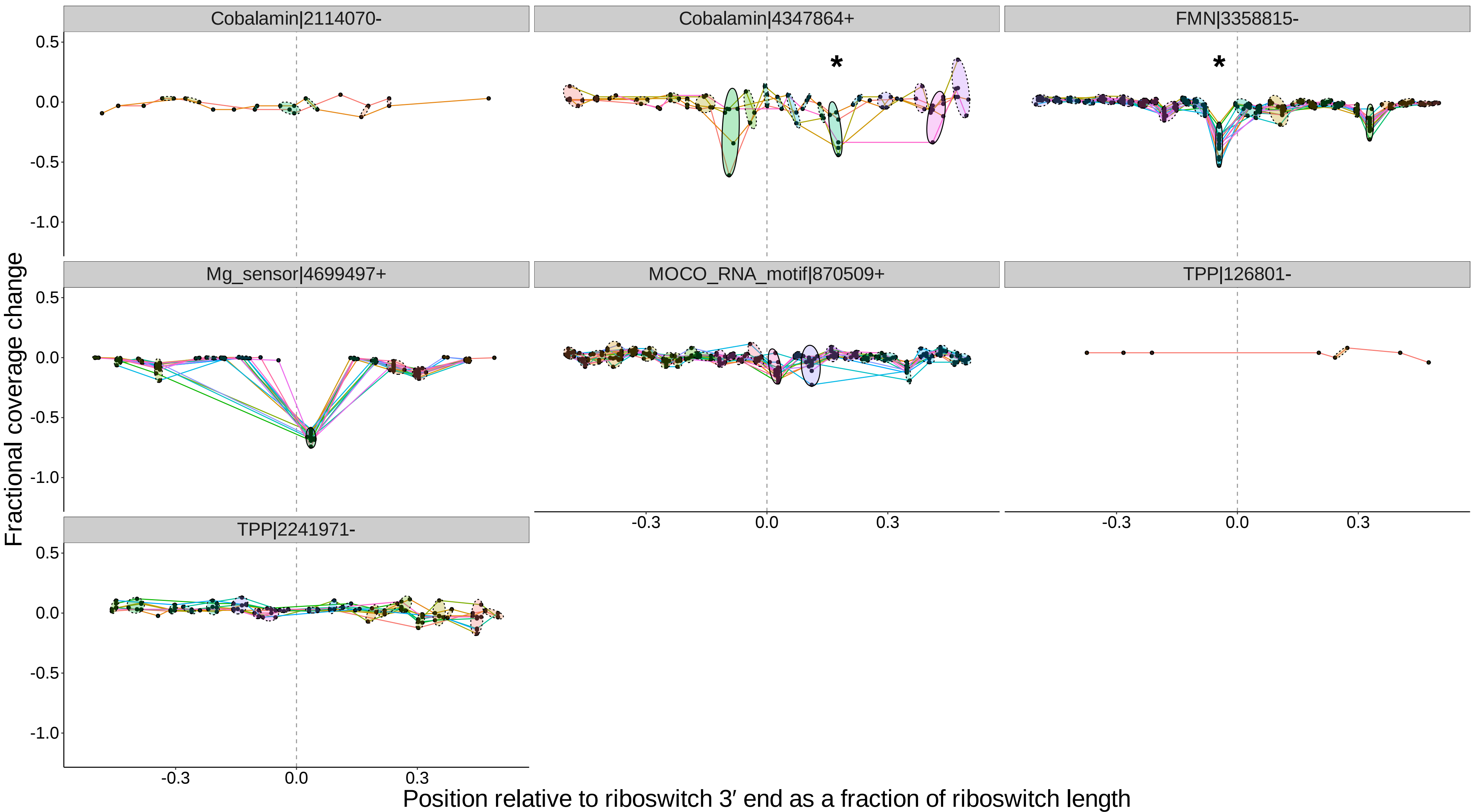

Supplement: Supplemental Information 7 — Peak clusters are grouped by colored ellipses; clusters with at least one significant transcription-termination peak are bounded by solid ellipses, and those with no significant peaks are bounded by dashed ellipses. Asterisks mark peak clusters that (i) contain at least one significant transcription-termination peak, (ii) have significantly lower mean (p < 0.05, Mann–Whitney one-tailed U-test) than the set of all other peak clusters, and (iii) have significantly higher variance (p < 0.05, Levene’s test) when compared to randomly sampled sets of the same number of peaks. [file peerj-13-19418-s007.pdf]
